# Preparation of FeS_2_/TiO_2_ nanocomposite films and study on the performance of photoelectrochemistry cathodic protection

**DOI:** 10.1038/s41598-021-87132-y

**Published:** 2021-04-05

**Authors:** Ning Wang, Jing Wang, Mengnan Liu, Chengyue Ge, Baorong Hou, Nazhen Liu, Yanli Ning, Yiteng Hu

**Affiliations:** 1 CAS Key Laboratory of Marine Envirinmental Corrosion and Bio-Fouling, Institute of Oceanology, Chinese Academy of Sciences, No.7 Nanhai Road, Qingdao, 266071 People’s Republic of China; 2 Open Studio for Marine Corrosion and Protection, Qingdao National Laboratory for Marine Science and Technology, Qingdao, 266237 People’s Republic of China; 3 Center for Ocean Mega-Science, Chinese Academy of Sciences, No.7 Nanhai Road, Qingdao, 266071 People’s Republic of China

**Keywords:** Materials science, Nanoscience and technology

## Abstract

FeS_2_/TiO_2_ nanotube array composite films with clean, high efficiency, low cost and low consumption were prepared by electrochemical anodization and hydrothermal methods. The modification of FeS_2_ nanoparticles on the surface of TiO_2_ nanotube array film not only broadens the light absorption range of TiO_2_, but also improves the utilization ratio of visible light and the separation rate of photogenerated electron–hole pairs, which greatly improves the photoelectrochemical cathodic protection performance of TiO_2_ for 304 stainless steel (304SS). Under visible light irradiation, the open circuit potential of 304SS coupled with the FeS_2_/TiO_2_ nanocomposite films decreased from − 170 to − 700 mV, and the electrode potential can still maintained at − 400 mV after the light was turned off. Compared with pure TiO_2_ nanotube array film, FeS_2_/TiO_2_ nanocomposite film has better photoelectrochemical cathodic protection effect on 304SS in 3.5 wt% NaCl corrosion medium.

## Introduction

Marine resources are rich, but the environment is complex and changeable, so many problems will be encountered in the process of marine development, and one of the most serious problems is marine corrosion. In marine engineering construction, 304SS is widely used because of its corrosion resistance. However, the high concentration of Cl^-^ in seawater makes 304SS prone to pitting corrosion, which leads to safety accidents. The traditional corrosion protection methods of 304SS include organic polymer coating, multi-layer composite coating, electrochemical cathodic protection, etc.^1–3^, but these technologies have the problems of high cost, short service life and environmental pollution. In recent years, more and more attention has been paid to photoelectrochemistry cathodic protection by coupling n-type semiconductors with metal materials.

The new photoelectrochemistry cathodic protection technology can improve the corrosion resistance of metal corrosion environment by converting solar energy into electrochemical energy, and in terms of material anti-corrosion, it has the advantages of strong operability, cleaning, resource saving and no power consumption. The application of semiconductor materials in the field of photoelectrochemical cathodic protection needs to meet certain conditions. Firstly, the conduction band potential of semiconductors must be lower than the self etching potential in the same solution; secondly, the band gap of semiconductors should not be too wide; thirdly, the photogenerated electron–hole pairs of semiconductors are easy to separate and have low recombination rate.

TiO_2_ is an n-type semiconductor material with excellent photoelectric performance, stable physical and chemical properties, low toxicity, rich resources and low manufacturing cost, and it has been widely used in the field of photoelectrochemistry cathodic protection^4–8^. However, the band gap of TiO_2_ is wide (3.2 eV), which can only absorb ultraviolet light with wavelength less than 378 nm, and has a low utilization rate of visible light; moreover, the electron–hole pair excited by light is easy to compound, and the efficiency of light quantum is low; furthermore, TiO_2_ can not play the role of photoelectrochemistry cathodic protection^9^ under dark conditions. Therefore, modification is an important research direction to improve the photoelectric conversion performance of TiO_2_. The modification methods of TiO_2_ mainly include: metal element doping (Ni^10^, Fe^11^, Zn^12^), nonmetallic element doping (N^13^, S^14^), and narrow band gap semiconductor sensitization (CdTe/TiO_2_^15^, CdSe/TiO_2_^16^, ZnSe/TiO_2_^17^, Bi_2_S_3_/TiO_2_^18^) and so on. Through modification, not only the band gap width of TiO_2_ can be reduced, but also the separation efficiency and photoelectrochemical properties of electron hole pairs can be improved, so as to improve the photoelectrochemistry cathodic protection effect of 304SS^19^.

FeS_2_ is widely distributed in nature, mainly in the form of hematite and pyrite. Hematite type has no photoelectric property, while it will change to pyrite type when the temperature exceeds 350 °C; pyrite FeS_2_ is a kind of non-toxic, stable physical and chemical properties, low cost, easy to obtain semiconductor materials, and it has appropriate band gap width (0.95 eV indirect band gap, 1.03 eV direct band gap width), and high light absorption coefficient (6 × 10^–5^ cm^−1^)^20^, which make FeS_2_ is useful for the preparation of optical devices that can utilize visible light. Han^21^ et al. prepared wormlike FeS_2_/TiO_2_ nanotube composites, which were used for photocatalytic reduction of CO_2_ to methanol under visible light, and the doping and recombination of FeS_2_ nanoparticles on TiO_2_ nanotubes not only broadened the light absorption range of TiO_2_, but also improved the photocatalytic reduction ability, which increased the methanol yield to 91.7 μmol/h/L. Rashid^22^ and others prepared stable anatase TiO_2_ composite pyrite FeS_2_ composite nanocrystals to improve the photocatalytic decomposition of methyl blue, and the catalytic decomposition of organic compounds increased from 15 to 75 mg/L within 180 min. C Mutalik^23^ et al. successfully prepared heterostructured TiO_2_-FeS_2_ nanocomposites (NCs) by a facile solution approach to enhance light-induced antibacterial activity over a broader absorption range, and the result showed that TiO_2_-FeS_2_ NCs had better antibacterial activity than that of only TiO_2_ nanoparticles (NPs) or only FeS_2_ NPs, which suggests that TiO_2_-FeS_2_ NCs with superior light-induced antibacterial activity could be a promising antibacterial agent against bacterial infections. Li et al.^24^ prepared FeS_2_ nanoparticles by a simple hydrothermal method. The FeS_2_ nanoparticles obtained by reducing FeCl_3_·6H_2_O with C_5_H_10_NS_2_Na·3H_2_O were assembled on nickel foam as the anode and C nanoparticles as the cathode, and then placed together in a double-electrode alkaline electrolytic cell. Kuo^25^ and others prepared a kind of clean hydrogen production based on TiO_2_ and environmentally friendly light catalyst by wet chemical synthesis method. The catalyst is composed of FeS_2_–TiO_2_ heterostructure nanocrystals, and its absorption range is wide, which can be extended from UV–Vis spectrum to near-infrared spectrum. A wider absorption wavelength range can improve the photocatalytic hydrogen production rate. Xin et al.^26^ prepared photoanode materials by sensitizing TiO_2_ nanotubes with FeS_2_ of pyrite. Due to the heterogeneous structure formed at the interface between FeS_2_ and TiO_2_, the recombination rate of photogenerated electron–hole pairs was reduced, and the light absorption capacity of TiO_2_ increased from the ultraviolet region to the near-infrared region, thus improving the photochemical properties of composite nanomaterials.

However, there is no report on the application of FeS_2_ particles modified TiO_2_ composite nanotube arrays for photoelectrochemical cathodic protection of 304SS. In this study, FeS_2_/TiO_2_ nanocomposite films were prepared by electrochemical anodization and hydrothermal methods to improve the optical absorption capacity of TiO_2_ in visible light and the photoelectrochemical cathodic protection performance, and the protection mechanism for 304SS has also been studied.

## Material and methods

### Preparation of TiO_2_ nanotube array films

Firstly, cut the industrial titanium plate (20 mm × 10 mm × 0.3 mm) with purity greater than 99.6%, and ultrasonic cleaning with acetone, absolute ethanol and ultrapure water for 10 min, separately, then make a mixture of 0.9 g ammonium fluoride, 5 mL ultra pure water, 12 mL hydrogen peroxide and 12 mL concentrated nitric acid to polish the titanium plate; secondly, platinum plate was used as counter electrode and titanium plate as anode, then put it in 80 mL ethylene glycol solution containing 0.44 g NH_4_F and 8 ml H_2_O and then it was anodized at a constant potential of 20 V for 1 h; thirdly, wash the obtained materials with deionized water and calcine it in a muffle furnace at 450 °C for 2 h to get TiO_2_ nanotube array films, then take it out and place in a dust-free dryer for standby.

### Preparation of FeS_2_/TiO_2_ nanocomposite films

FeS_2_/TiO_2_ nanocomposite films were prepared by hydrothermal deposition of FeS_2_ nanoparticles on TiO_2_ nanotubes. The detailed process is as follows: Firstly, FeCl_3_·9H_2_O was dissolved with secondary water in a 100 mL beaker, and prepare 35 mL FeCl_3_ solutions with concentrations of 5 mmol/L, 10 mmol/L and 20 mmol/L respectively, then dissolve thiourea ((NH_2_)_2_CS) with secondary water in another beaker to make Fe:S = 1:2, and then stir the two solutions for 30 min respectively and then set aside; Secondly, add 35 mL thiourea solution into FeCl_3_ solution and stir for 20 min after dropping to make FeCl_3_ and thiourea fully mixed; thirdly, transfer the mixed solution to a 100 mL Teflon reactor containing TiO_2_ nano film sheet, then placed it in the stainless steel jacket and put it into the blast drying oven, and set the heating temperature as 180 °C and heating time as 12 h; Finally, after the autoclave is cooled naturally, wash the obtained samples with deionized water and ethanol, and dried with dry cold air, then mark the obtained samples as 5-FST, 10-FST and 20-FST respectively for standby.

### Characterization

The morphology of pure TiO_2_ and FeS_2_/TiO_2_ nanocomposite films were observed by scanning electron microscopy (SEM, Hitachi, S-8100); the chemical composition of FeS_2_/TiO_2_ nanocomposites was measured by X-ray photoelectron spectroscopy (XPS, PHI, Quantum 2000); and the crystal structures of TiO_2_ and FeS_2_/TiO_2_ composites were detected by X-ray diffraction (XRD, Japan Science and technology company, 5°/min); and the optical absorption properties of TiO_2_ and FeS_2_/TiO_2_ composites were characterized by uv-drs (Hitachi).

### Photoelectrochemistry cathodic protection performance measurement

**Figure 1:**
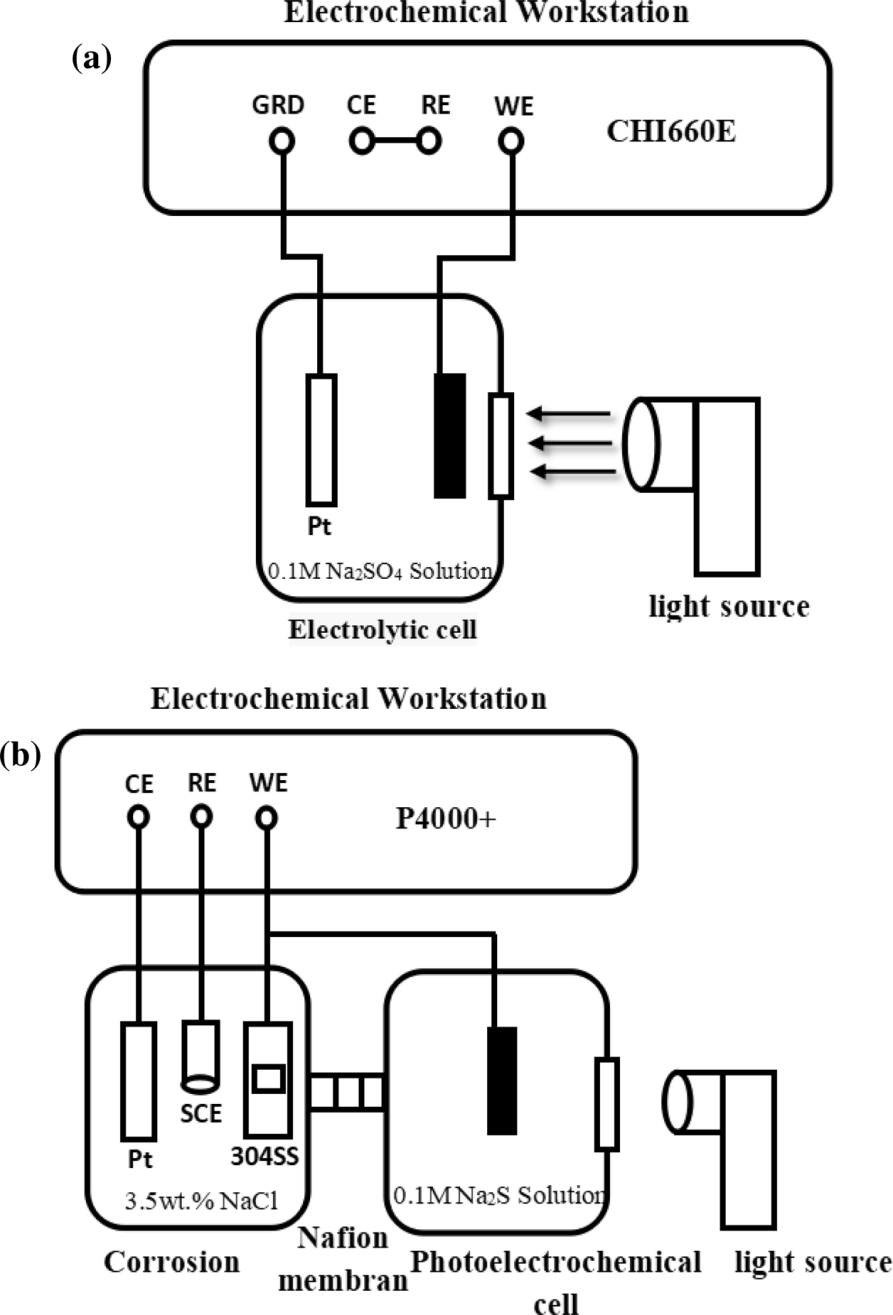
(**a**) Photocurrent density measurement setup, (**b**) Experiment setup for electrochemical measurement.

The photoelectrochemical properties are evaluated by measuring the photocurrent density time curve (I–t), and the test device is shown in Fig. 1a. The reference electrode and counter electrode of the electrochemical workstation CHI660E are short circuited, and a small resistance ammeter is formed between the working electrode and the grounding end. The electrolyte in the electrolytic cell is 0.1 mol/L Na_2_SO_4_, and the light source is the incident light filtered out of ultraviolet rays. As shown in Fig. 1b, the device for testing the performance of photoelectrochemical cathodic protection (including OCP, Tafel and EIS) is a dual cell system consisting of a corrosion cell containing 3.5 wt% NaCl solution and a photocell containing 0.1 mol/L Na_2_S solution, and the two cells are connected by Nafion membrane. The electrochemical work station (P4000 + ,USA) of three electrode system was used for electrochemical measurement: platinum plate as counter electrode, saturated calomel electrode as reference electrode, and copper wire as working electrode between 304SS electrode and optical electrode. The light source is a 300 W high pressure xenon lamp with visible light wavelength greater than 400 nm.

## Results and discussion

### Analysis of open circuit potential time curve

**Figure 2:**
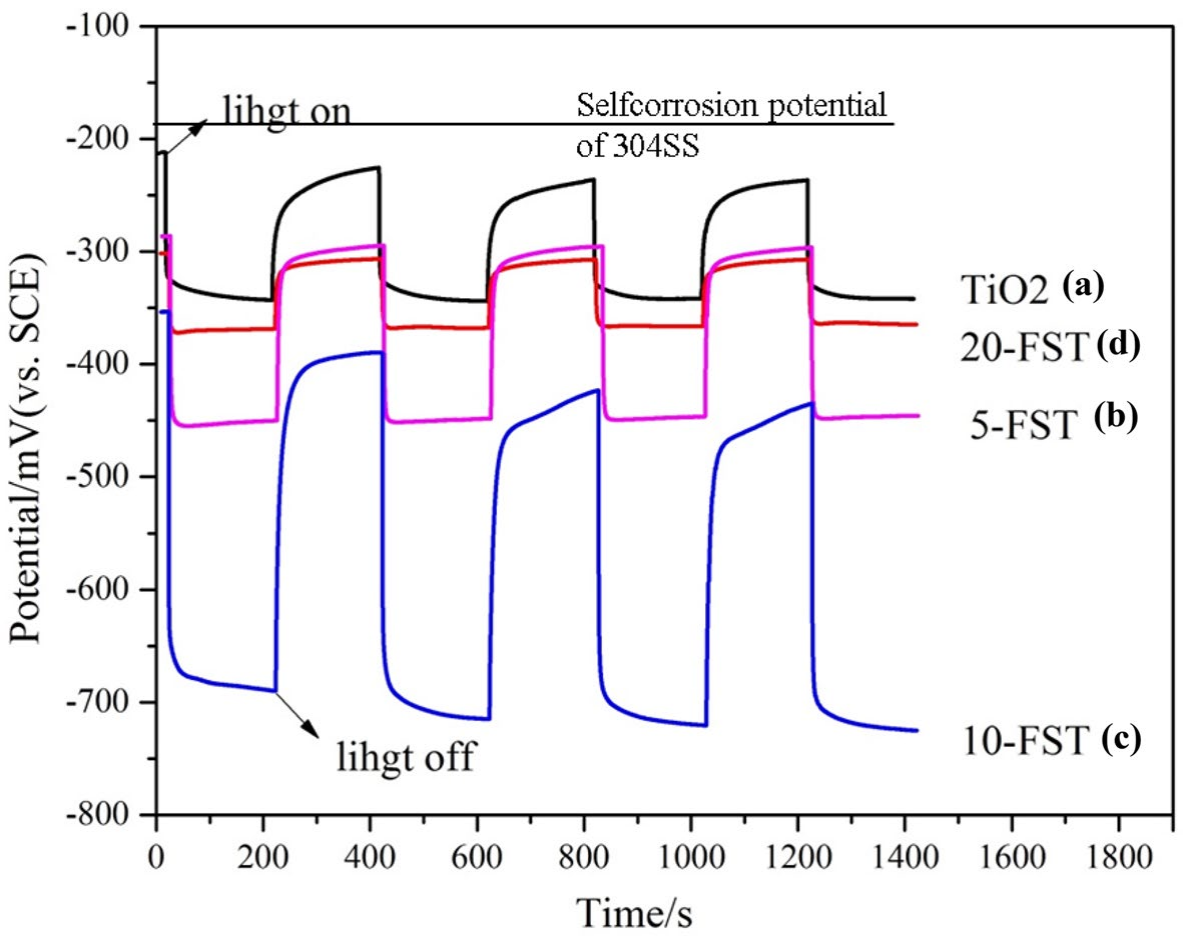
OCP of (**a**) pure TiO_2_, (**b**) 5-FST, (**c**) 10-FST, (**d**) 20-FST.

Figure 2 shows the open circuit potential time curves of 304SS electrode, 304SS electrode coupled with pure TiO_2_ nanotube array film, and 304SS electrode coupled with three kinds of FeS_2_/TiO_2_ nanocomposite films as photoanode under intermittent visible light and in 3.5 wt% NaCl electrolyte, and the OCP reflects the corrosion state of stainless steel electrode. It can be seen from the figure that the self corrosion potential of 304SS in 3.5 wt% NaCl corrosion medium is − 170 mV. As shown in curve (a), the electrode potential of 304SS coupling with pure TiO_2_ sample decreased from − 210 to − 340 mV under visible light, and the electrode potential after reaching stability was more negative than that of 304SS (− 170 mV), because TiO_2_ photo anode provided power supply for 304SS electrode under light condition, which reduced the electrode potential, thus realizing the effect of photoelectrochemical cathodic protection; when the light stopped, the OCP returned to the coupling electrode potential before the light, which indicated that pure TiO_2_ had no photoelectrochemical cathodic protection effect on 304SS electrode in the dark. Curves (b-d) show the OCP of 304SS coupling samples 5-FST, 10-FST and 20-FST as photoanodes, and the electrode potential of 304SS with three kinds of FeS_2_/TiO_2_ nanocomposite films decreased to − 455 mV, − 708 mV, − 361 mV under visible light, respectively, which indicates that the deposition of FeS_2_ nanoparticles on the surface of TiO_2_ nanofilms can improve the photoelectrochemical properties of TiO_2_. With the increase of reactant concentration in hydrothermal reaction, the photoinduced potential drop first increases and then decreases, and the photochemical cathodic protection ability also increases first and then decreases. Combined with the SEM surface morphology in Fig. 4, it is speculated that the reason may be that the excessive FeS_2_ nanoparticles not only reduce the active sites for light energy absorption of TiO_2_ films, but also increase the recombination sites of photoinduced electron hole pairs. When the light stopped, the electrode potential of 304SS coupled with three samples increased to − 320 mV, − 390 mV and − 310 mV, respectively, however, these three potentials are lower than that of 304SS bare electrode, which indicates that FeS_2_/TiO_2_ nanocomposite film still has good photoelectrochemistry cathodic protection effect under dark conditions, and the reason may be FeS_2_/TiO_2_ nanocomposite materials having certain energy storage function.

### Analysis of photocurrent density time curve

**Figure 3:**
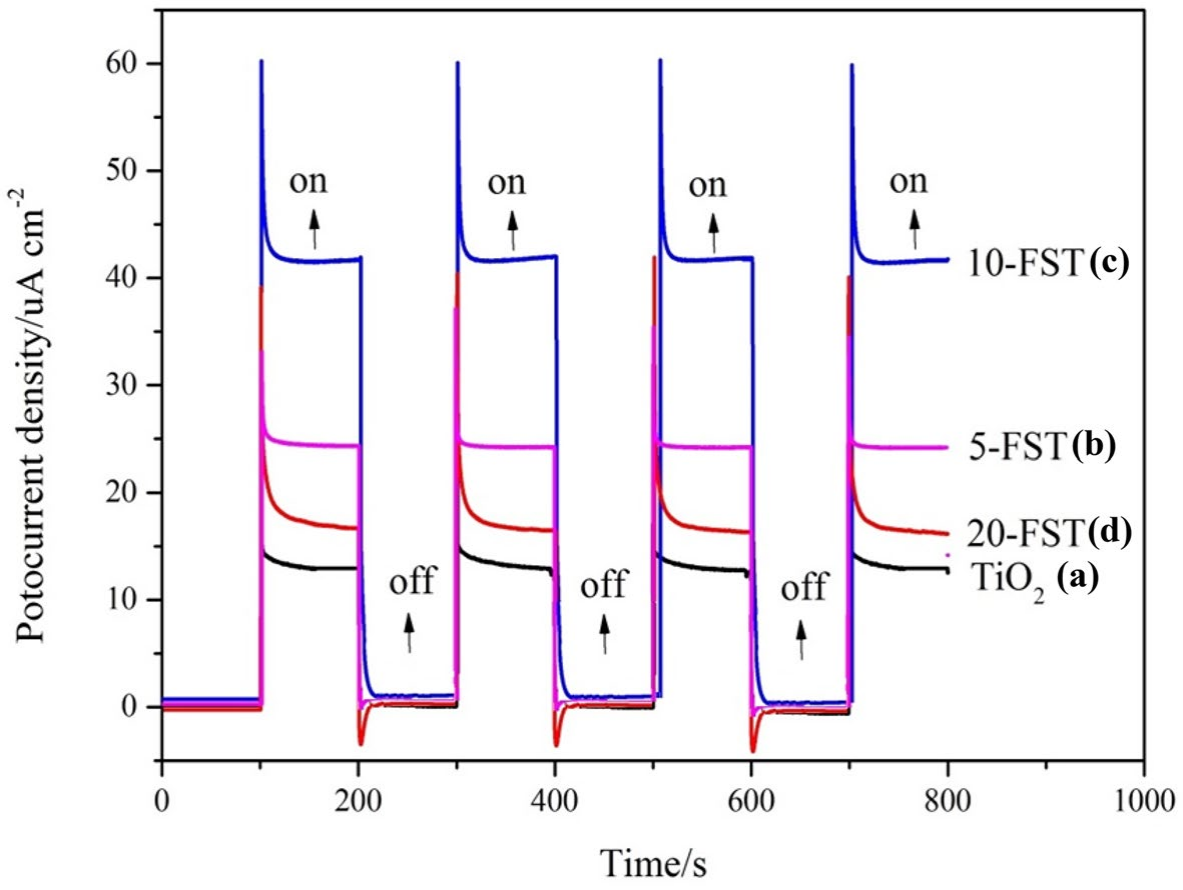
Photocurrent density time curve of TiO_2_ (**a**), 5-FST (**b**), 10-FST (**c**), 20-FST (**d**).

Figure 3 shows the time curve of photocurrent density measured under intermittent visible light and 0.1 mol/L Na_2_SO_4_ solution by connecting TiO_2_ nanotube array film and FeS_2_/TiO_2_ composite film prepared at different concentrations as photoanode. The photocurrent density time curve can directly show the photoelectric conversion efficiency of photoelectric materials. The higher the photocurrent density, the higher the photoelectric conversion efficiency, and the stronger the metal protection. As shown in the figure, when the visible light irradiates the surface of the materials, the photocurrent provided by these materials as photoanodes to the cathode increases instantaneously and then stabilizes at a constant value, indicating that they all have photoelectric response performance. It can be seen from curve (a) that a very small photocurrent density (13 μA/cm^2^) is generated by the TiO_2_ photoanode, which is due to the large band gap of pure TiO_2_ and the small light absorption in the visible region. The curve (b-d) show that the current densities provided by the three kinds of FeS_2_/TiO_2_ composite films are 25 μA/cm^2^, 43 μA/cm^2^ and 18 μA/cm^2^, respectively, which are higher than those of pure TiO_2_ nano film as photoanode. The results show that the modification of TiO_2_ nano film by FeS_2_ nanoparticles greatly improves the photoelectric conversion efficiency, which is due to FeS_2_ semiconductor with narrow band gap is easy to absorb visible light, and the photo excited electrons migrate from the FeS_2_ conduction band to the TiO_2_ conduction band, while the photogenerated holes gather in FeS_2_, which realizes the separation of photogenerated electron–hole pairs, thus improving the light utilization and conversion efficiency of TiO_2_^27^. Similar to pure TiO_2_ nanotube array film, many experiments on opening and closing light show that FeS_2_/TiO_2_ nanocomposite films have very high stability and can not be corroded by light.

### Morphology characterization

**Figure 4:**
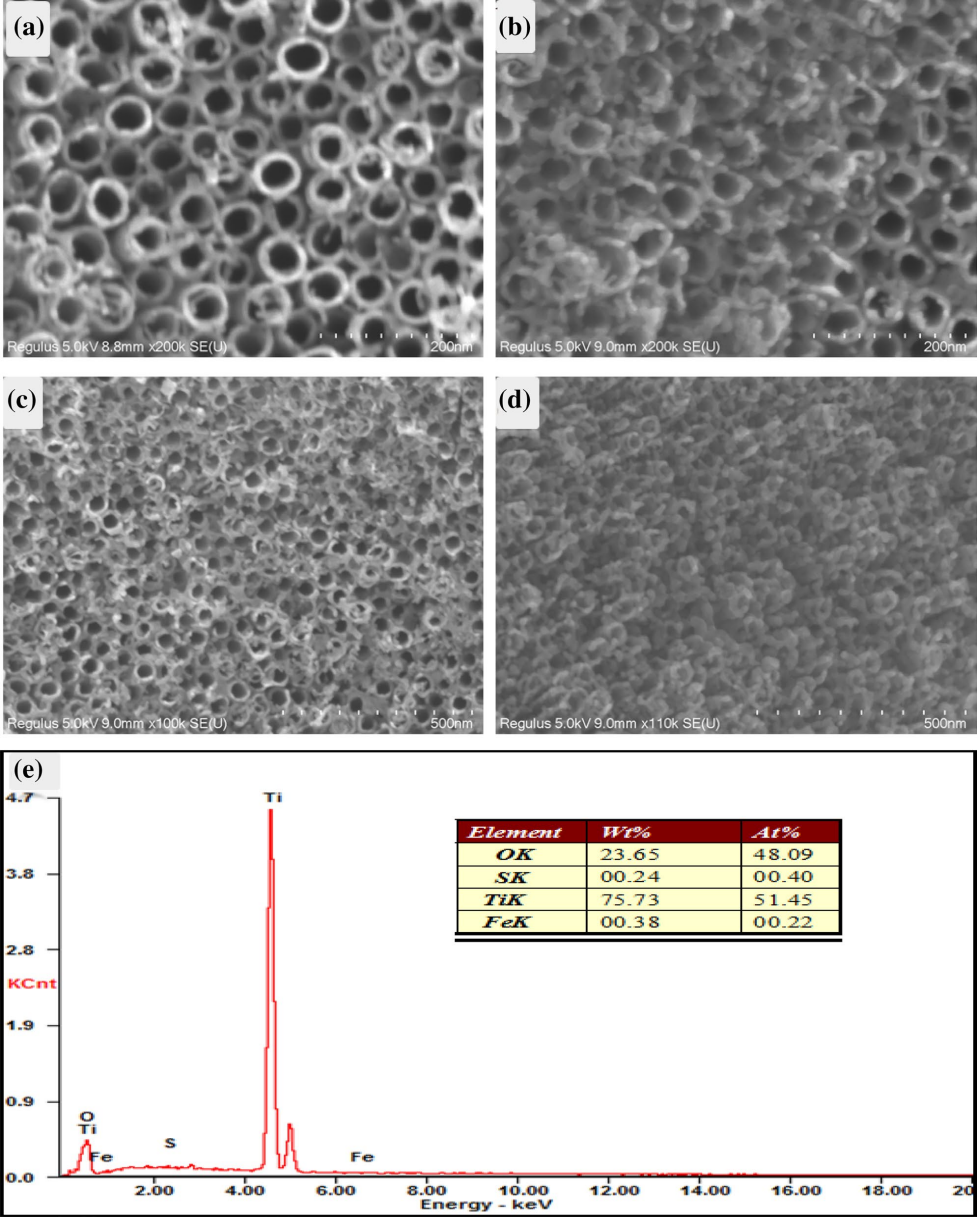
SEM images of (**a**) pure TiO_2_, (**b**) 5-FST, (**c**) 10-FST, (**d**) 20-FST; (**e**) EDS of 10-FST.

The surface morphology of the samples is shown in Fig. 4, among them, Fig. 4a shows the SEM of pure TiO_2_, Fig. 4b–d shows the SEM of 5-FST, 10-FST and 20-FST, respectively. It can be seen from Fig. 4a that the tubular structure is formed by electrochemical oxidation, and these nanotubes whose diameter is 60–90 nm are closely arranged to form a thin film covering the smooth surface. The thickness of the tube wall is about 15–20 nm, and the length of the nanotube is about 450 nm (as shown in the cross-section at the upper right corner). Figure 4b is the SEM image of 5-FST sample, and it can be seen that nanoparticles with a diameter of 10–20 nm are attached to the edge of the tube mouth, and there is no deformation or collapse of TiO_2_ nanotubes after hydrothermal reaction, which indicates that the thermodynamic properties of TiO_2_ crystal are very stable. Figure 4c is the morphology of 10-FST sample, and it can be clearly observed that with the increase of FeS_2_ nanoparticles, the coverage area of FeS_2_ on the TiO_2_ nanotube film expands, and gradually agglomerates on the surface of TiO_2_ then fills the nanotubes, but the nozzle of TiO_2_ is still open. The morphology of 20-FST sample is shown in Fig. 4d, and the large FeS_2_ particles almost seal the mouth of TiO_2_ film. In conclusion, with the increase of the concentration of hydrothermal deposition reactants, the deposition amount of FeS_2_ nanoparticles increases, while the open part of TiO_2_ nanotubes decreases, as well as the contact area of photoelectrochemical reaction decreases, which indicates that the concentration of reactants will affect the morphology of FeS_2_/TiO_2_ nanocomposite films. Figure 3e is the energy spectrometer point distribution diagram of FeS_2_/TiO_2_ nanocomposite films. There is not only a large amount of Ti, O elements, but also a small amount of Fe and S elements were detected, and the ratio of Fe to s is about 1:2. For the analysis of the specific composition and the compound state of the substance, we will further determine it by means of X-ray photoelectron spectroscopy.

### Crystal structure analysis of FeS_2_/TiO_2_ nanocomposite films

**Figure 5:**
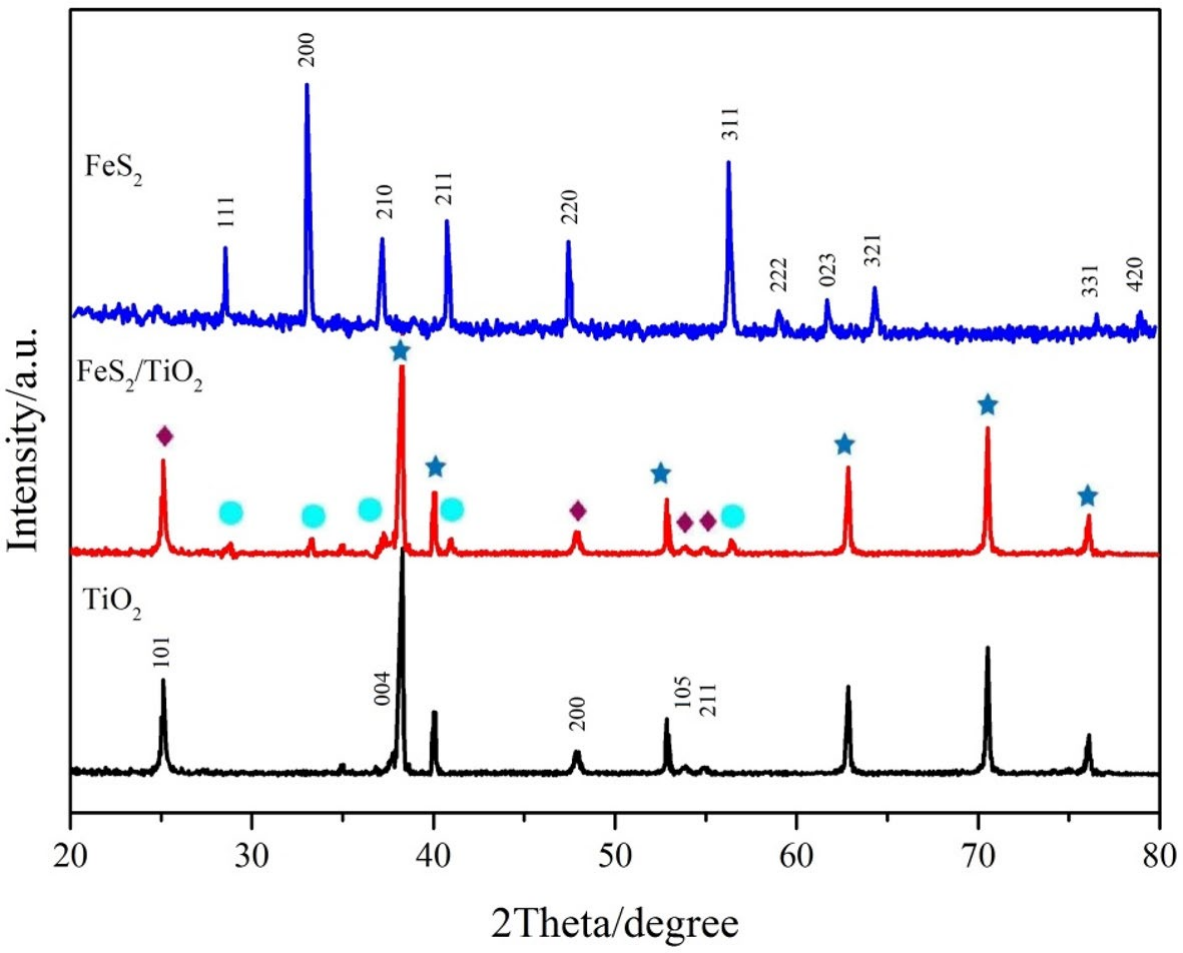
XRD patterns of pure TiO_2_, pure FeS_2_ and FeS_2_/TiO_2_ nanocomposites, the rhombus represents anatase type of TiO2 and star represents rutile type of TiO2.

Figure 5 shows the XRD spectra of pure TiO_2_ nanotube array film sample, FeS_2_/TiO_2_ nanocomposite film sample and pure FeS_2_ powder prepared by hydrothermal method. As shown in the XRD curve of pure TiO_2_, there are two groups of characteristic diffraction peaks in the sample. A group of characteristic diffraction peaks with 2θ at 25.3°, 37.8°, 48.0°, 53.9° and 55.0° respectively belong to anatase TiO_2_ (JCPDF-21-1272), and these five diffraction peaks correspond to the 101, 004, 200, 105, 211 crystal face of TiO_2_, among them, 101 crystal face diffraction peak is the strongest, which indicates that 101 crystal face has the strongest activity; the 2 θ of the other group of characteristic diffraction peaks is 38.4°, 40.2°, 53.0°, 62.9°, 70.7° and 77.4° respectively, which is the characteristic diffraction peak of Ti metal lattice (JCPDF-44-1294), and the diffraction peak intensity of Ti is much stronger than that of TiO_2_, which verifies that the electrochemical oxidation of TiO_2_ is a nano material; while there is no diffraction peak of rutile TiO_2_ on the curve of this sample, which indicates that the crystal structure of calcined samples is anatase. In the XRD curves of FeS_2_/TiO_2_ nanocomposite film, in addition to the characteristic diffraction peaks of pure TiO_2_, there are also characteristic absorption peaks at 28.52° 33.05° 37.07° 40.75° 56.23° etc., which indicates that pyrite FeS_2_ nanoparticles were successfully deposited on anatase TiO_2_ nanofilms to form FeS_2_/TiO_2_ nanocomposites, which was consistent with the results of SEM. From the XRD spectra of FeS_2_ powder, it can be seen that the characteristic diffraction peak appears with 2θ at 28.51°, 33.04°, 37.07°, 40.76°, 47.42°, 50.49°, 56.27°, 59.01°, 61.68°, 64.28°, 76.59° and 78.96° respectively, and corresponding to (111), (200), (210), (211), (220), (311), (222), (023), (321), (331), and (420) of pyrite and crystal face (JCPDF-42-1340), respectively^25^, and there are no other impurity diffraction peaks in the curve, which indicates that the preparation method used in this study can prepare pyrite FeS_2_ nanoparticles with high purity and crystallinity.

### XPS analysis of FeS_2_/TiO_2_ nanocomposite films

**Figure 6:**
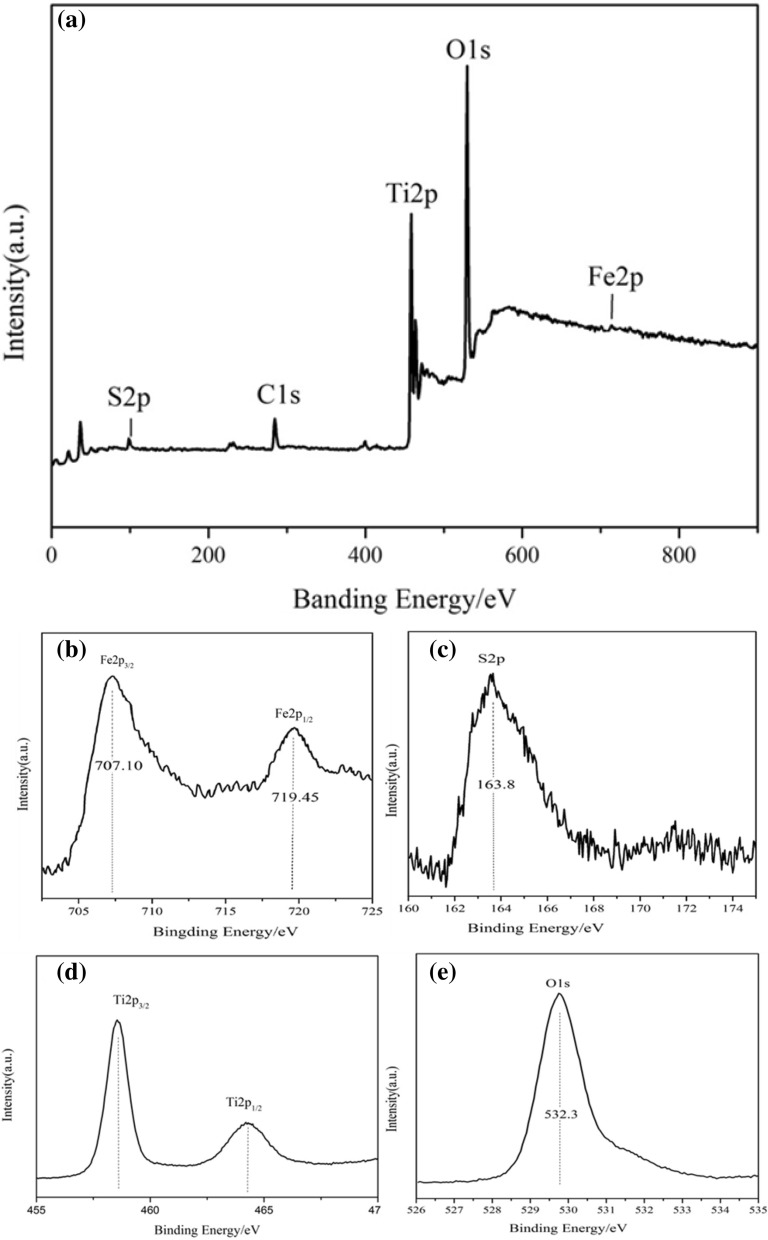
XPS survey spectra (**a**), Fe2p (**b**), S2p (**c**), Ti2p (**d**), O1s (**e**) core level spectrum of FeS_2_/TiO_2._

In order to confirm the composition of the nanocomposite films more clearly, we used X-ray photoelectron spectroscopy to detect the valence orbital structure and valence of elements. Figure 6 shows the XPS spectra of FeS_2_/TiO_2_ nanocomposite film prepared under hydrothermal reaction time of 12 h, ferric chloride concentration of 0.15 mmol/L and reaction temperature of 180 °C, among them, Fig. 6a is the full spectrum scanning, and Fig. 6b–e is the high resolution narrow spectrum scanning map of elements. It can be seen from Fig. 6a that the characteristic orbital absorption peaks of Fe2p, O1s, Ti2p, S2p and C1s are detected in the sample, and there are no absorption peaks of other elements in the full spectrum, while the peak of C1s is the impurity peak when the instrument is calibrated. Figure 6b is a high resolution narrow spectrum of Fe2p, and it can be seen that the binding energies at 707.1 eV and 719.4 eV correspond to the orbits of Fe2p_3/2_ and Fe2p_1/2_, and the distance between the two peaks is 12 eV, which is similar to that of FeS_2_. Figure 6c is a high-resolution narrow spectrum of S2p orbit, and 163.8 eV corresponds to the characteristic peak of binding energy of S2p orbit. According to the literature, the S element in the sample is − 1 valence, so FeS_2_ exists in the complex^28^. Figure 6d is a high resolution narrow-band XPS image of Ti2p, and the binding energies of the two characteristic absorption peaks are 458.4 eV and 464.2 eV, respectively, which are corresponding to the orbital absorption peaks of Ti2p_3/2_ and Ti2p_1/2_, indicating that the valence state of Ti is + 4, and the Ti element in the sample mainly exists in the form of TiO_2_. Figure 6e is a high resolution narrow spectrum of O1s, and it can be seen from the figure that the characteristic peak of binding energy at 529.9 eV corresponds to the metal oxide, which is consistent with the narrow spectrum scanning of Ti2p, and due to the O1s peak is very sharp, there is no other oxide in the sample. In conclusion, the composition of the nanocomposite is only FeS_2_ and TiO_2_, which is consistent with the results of EDS and XRD.

### Optical absorption properties of FeS_2_/TiO_2_ nanocomposite films

**Figure 7:**
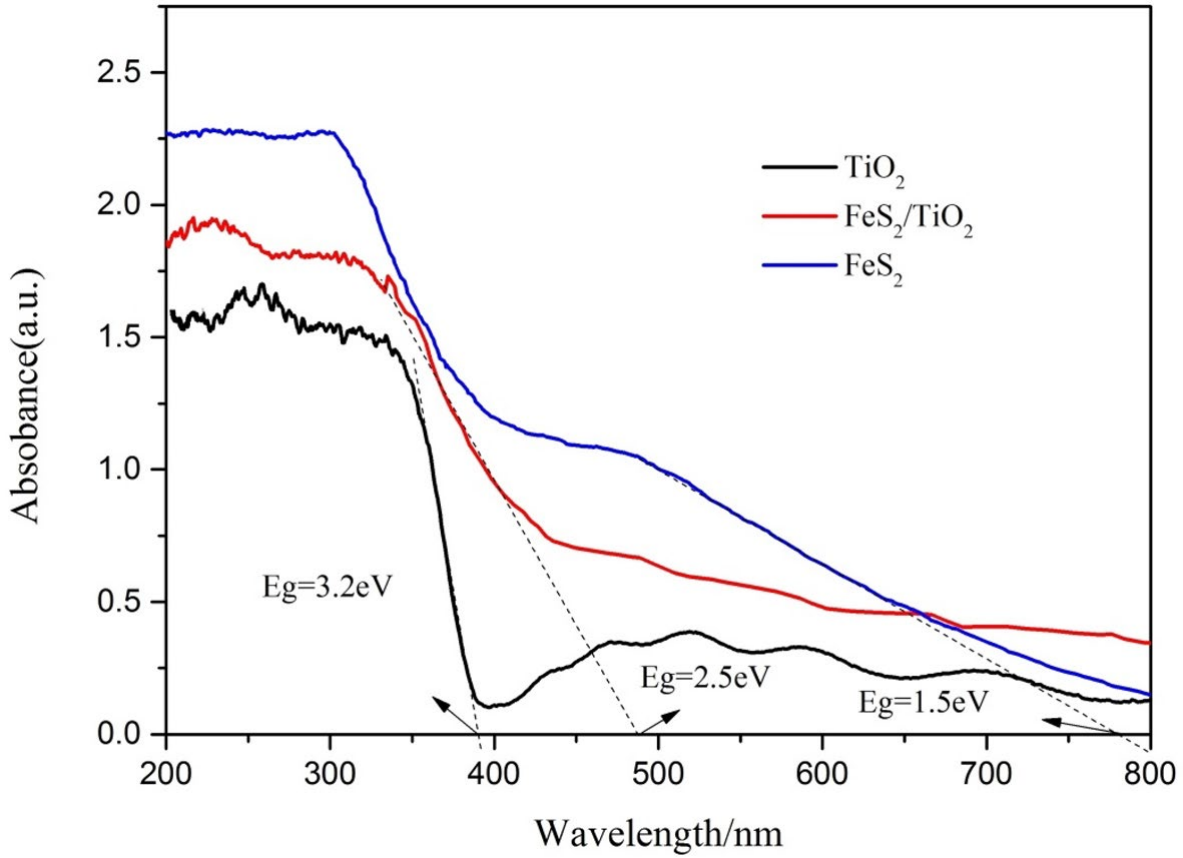
UV–vis diffraction reflectance spectra of the pure TiO_2_, pure FeS_2_ and FeS_2_/TiO_2._

Figure 7 shows the UV–Vis diffuse reflectance spectra of pure TiO_2_ nanotube film, pure FeS_2_ powder tablet and FeS_2_/TiO_2_ nanocomposite film. It can be seen from the curve of pure TiO_2_ sample that the light absorption edge of pure TiO_2_ nano film is 387 nm, and the light absorption intensity in visible light region is very weak, corresponding to the energy band width of TiO_2_ is 3.2 eV. From the curve of pure FeS_2_ sample we can see that the light absorption edge of pyrite FeS_2_ is 780 nm, and the radiation in visible spectrum has strong light response, and the corresponding band gap is 1.5 eV, indicating that pyrite FeS_2_ is an excellent semiconductor material with narrow band gap. According to the curve of FeS_2_/TiO_2_ nanocomposite films, the light absorption edge of FeS_2_/TiO_2_ nanocomposite is 496 nm, and the corresponding band gap is 2.5 eV, which indicates that the deposition of FeS_2_ nanoparticles on the TiO_2_ nano film can expand the light absorption range to the visible light region, and reduce the band gap of TiO_2_. In summary, nanosized pyrite FeS_2_ has a very significant effect on improving the optical absorption properties of TiO_2_ nanotube films.

### Tafel polarization curve analysis of FeS_2_/TiO_2_ nanocomposite films

**Figure 8:**
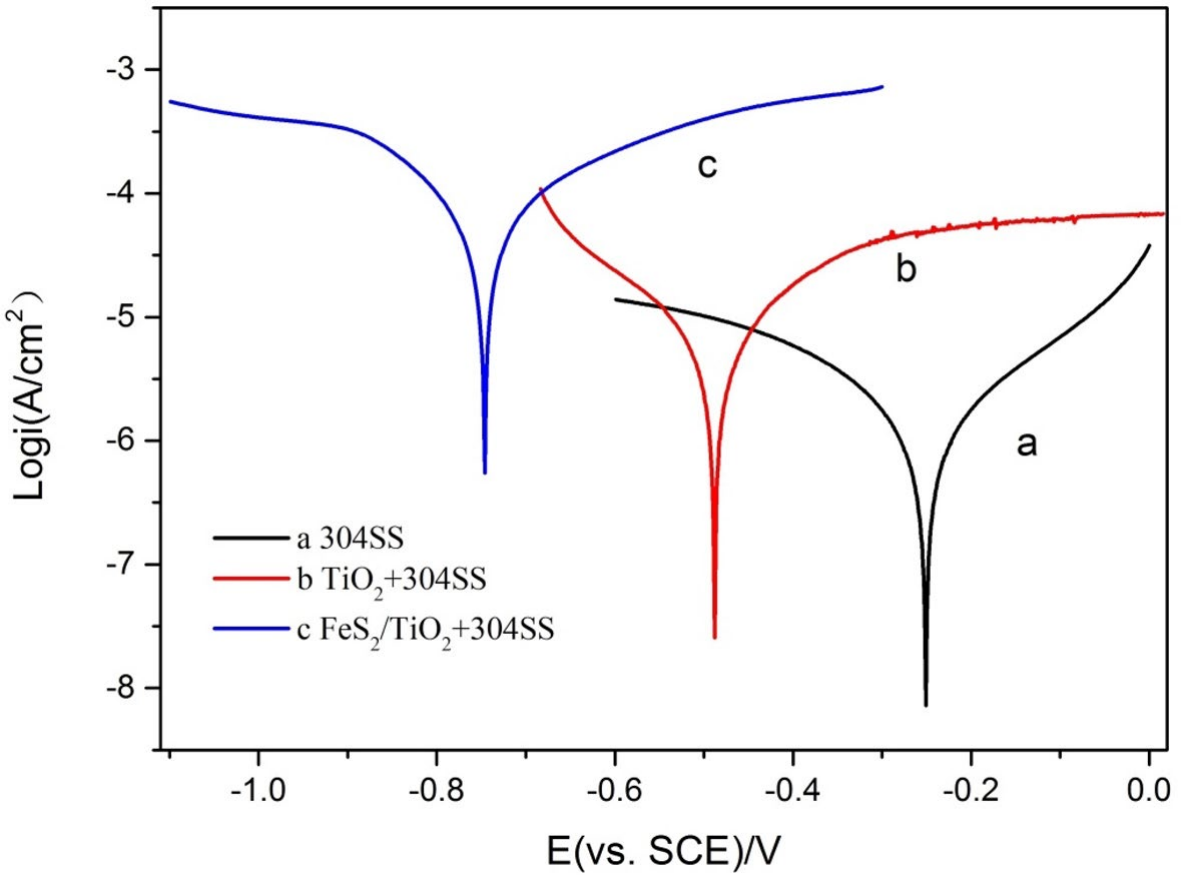
Tafel polarization curves of bare 304SS electrode (**a**), and 304SS coupled with pure TiO_2_ (**b**) and FeS_2_/TiO_2_ (**c**) under visible light illumination.

After the metal contacts with the medium in the environment, there are multiple electrode reactions on the metal surface. The anodic reaction makes the metal dissolve and enter the solution in the form of ions, which is called anode process; and the cathodic reaction is that the medium on the metal surface absorbs the electrons from the anode, and the metal does not dissolve. The existing state of metal depends on the addition of total cathodic reaction and total anode reaction, and the anode process causes electrochemical corrosion of metal, while the principle of cathodic protection is the polarization of metal under external conditions, therefore, the total potential of metal changes from corrosion potential to negative, the total cathodic current increases and the anode current decreases. Figure 8 shows the Tafel potentiodynamic polarization curve of 304SS electrode coupled with TiO_2_ nanotube array film and FeS_2_/TiO_2_ nanocomposite film photoanode under visible light condition, and the corrosion medium is 3.5 wt% NaCl solution. As shown in curve (a), the corrosion potential of 304SS bare electrode is − 180 mV in 3.5 wt% NaCl solution. It can be seen from the curves (b) and (c), under visible light irradiation, the potential of 304SS coupled with pure TiO_2_ and FeS_2_/TiO_2_ photoanode shifted negatively to − 490 mV and − 740 mV, separately, and the 304SS was in cathodic protection state. It can also be seen from the figure that the current on the surface of 304SS electrode coupled with pure TiO_2_ and FeS_2_ photoanode is larger than that of bare 304SS electrode, which indicates that the photoanode provides electrons for 304SS under visible light, and this result is consistent with the open circuit potential time curve and photocurrent density time curve. According to the principle of cathodic protection, the electrode potential of metal decreases, the current increases, and the electrode reaction of metal corrosion is inhibited, which shows that both pure TiO_2_ and FeS_2_/TiO_2_ can provide photoelectrochemical cathodic protection for 304SS under visible light. Compared with pure TiO_2_, FeS_2_/TiO_2_ has better cathodic protection effect on 304SS, which indicates that FeS_2_ modified TiO_2_ improves the photoelectrochemical cathodic protection performance of TiO_2_ nanotube array film.

### EIS analysis of FeS_2_/TiO_2_ nanocomposite films

**Figure 9:**
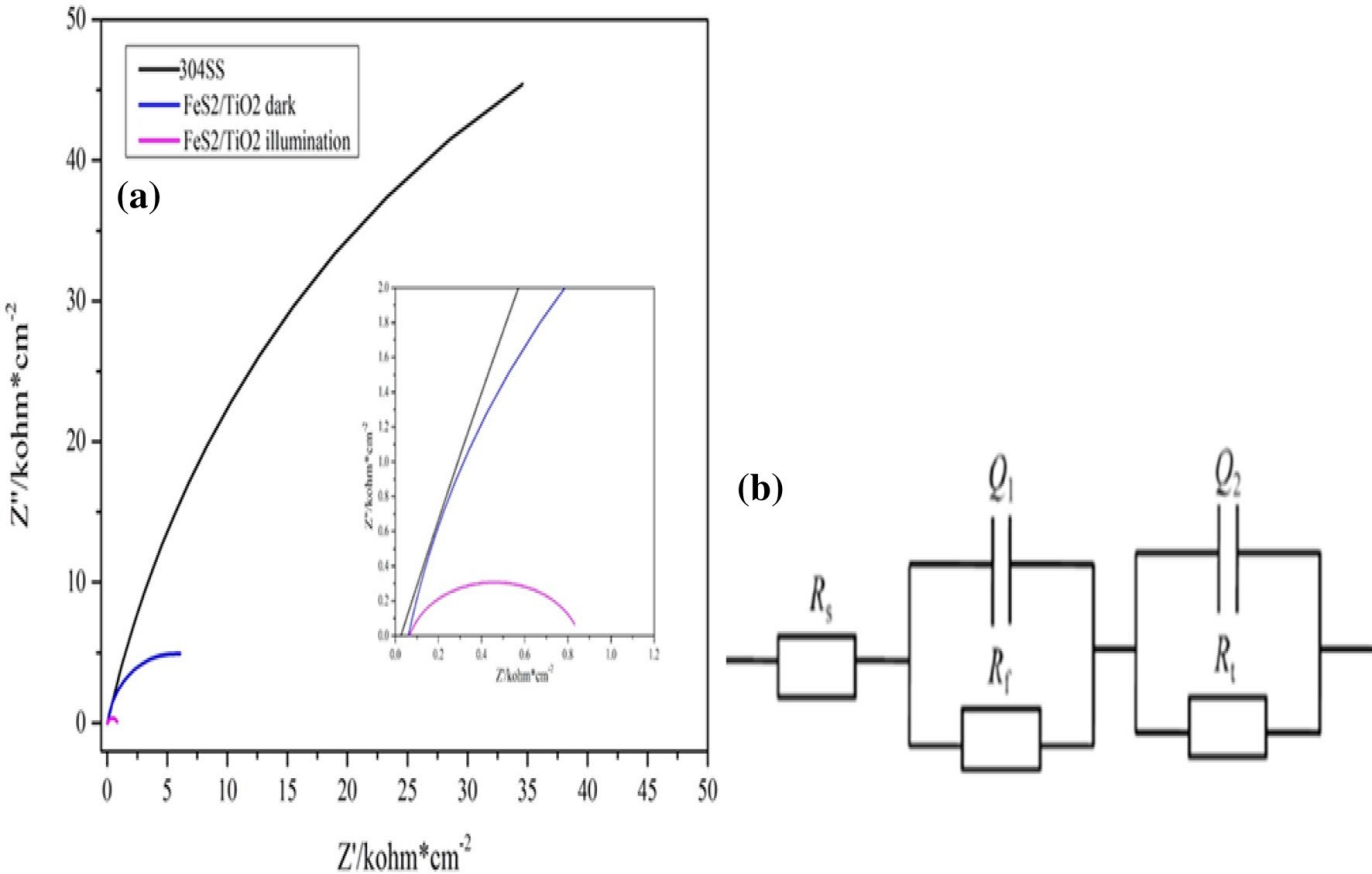
(**a**) Nyquist plots of 304SS, 304SS coupled with FeS_2_/TiO_2_ under visible light illumination and dark state conditions ; (**b**)The equivalent circuit model of FeS_2_/TiO_2_ photoanode under illumination.

EIS can provide valuable electrochemical information of metal/solution interface, so it is used to evaluable the effect of photochemical cathodic protection on the prepared films^29^. Figure 9a shows the Nyquist diagram of 304SS in 3.5 wt% NaCl solution without coupling and coupling FeS_2_/TiO_2_ nano film photoanode, and each curve has only one capacitance arc. Obviously, under visible light irradiation, the capacitance arc of 304SS coupling FeS_2_/TiO_2_ nano film photoanode is much smaller than that of 304SS bare electrode, and the difference between them is two orders of magnitude. The EIS spectrum can be simulated by the equivalent circuit shown in Fig. 9b. In the equivalent circuit model, RS, RCT and CPE represent solution resistance, charge transfer resistance and constant phase element respectively, and the main purpose of EIS measurement is to obtain the RCT value of coupled 304SS in corrosion cell to compare the electron transfer rate under light and dark conditions. When 304SS was coupled with thin film photoanode under light, photogenerated electrons were transferred from photoanode to steel to participate in electrochemical reaction, which accelerated the cathodic reaction of 304SS stainless steel/solution interface, showed low RCT value, and 304SS cathode polarization reduced corrosion. Therefore, FeS_2_/TiO_2_ nanocomposite films can provide excellent photochemical cathodic protection performance.

### Mechanism analysis of photoelectrochemical cathodic protection

**Figure 10:**
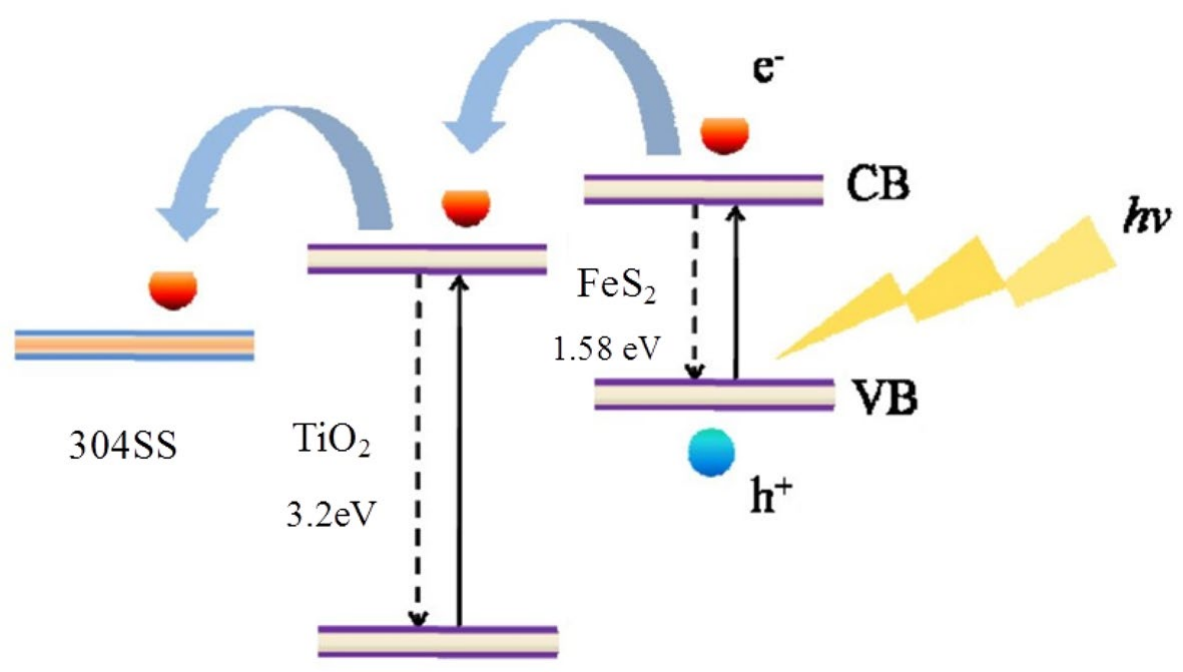
The mechanism schematic diagram of the photoelectochemical cathodic protection.

The electron transfer process of FeS_2_/TiO_2_ nanocomposite film coupled with 304SS electrode under visible light is shown in Fig. 10. It takes less photon energy to excite FeS_2_, and the electron in the valence band of FeS_2_ can be excited by visible light and then transited to the conduction band. Because the conduction band potential of FeS_2_ is lower than that of TiO_2_, the conduction band electrons in FeS_2_ can be transferred to the conduction band of TiO_2_, while the holes in FeS_2_ remain in the valence band, which greatly improves the separation efficiency of photogenerated electron–hole pairs. Under the action of external circuit, photogenerated electrons are transferred to 304SS and gathered on the surface of it, which makes 304SS cathode polarized to achieve the purpose of cathodic protection. Therefore, FeS_2_/TiO_2_ nanocomposite film has excellent photoelectrochemical cathodic protection effect on 304SS under visible light irradiation.

## Conclusion

1. FeS_2_/TiO_2_ nanocomposite films were successfully prepared by anodic oxidation and hydrothermal methods, and when the concentration of reactants was designed to be 10 mmol/L, FeS_2_/TiO_2_ nanocomposite films had the optimal surface morphology of absorbing sunlight.
2. After the modification of FeS_2_ nanoparticles, the light absorption range of TiO_2_ extends from UV to visible, which not only improves the separation efficiency of photogenerated electron–hole pairs, but also increases the photoelectric conversion ability.
3. Compared with pure TiO_2_, the modification of FeS_2_ nanoparticles on the surface of TiO_2_ nanotube array film greatly improves the photoelectrochemistry cathodic protection performance of 304SS.
4. Under visible light irradiation, the OCP of 304SS coupled with the composite can be reduced to about − 700 mV, and the electrode potential of the stainless steel can still be maintained at − 400 mV after the light is turned off, that is, FeS_2_/TiO_2_ Nanocomposite filmscan still play the role of electrochemical cathodic protection in the dark.
